# What Makes the Optimal Wound Healing Material? A Review of Current Science and Introduction of a Synthetic Nanofabricated Wound Care Scaffold

**DOI:** 10.7759/cureus.1736

**Published:** 2017-10-02

**Authors:** Matthew R MacEwan, Sarah MacEwan, Tamas R Kovacs, Joel Batts

**Affiliations:** 1 Research & Development, Acera Surgical, Inc; 2 Research, Telos Partners, Llc

**Keywords:** wound, wound care, chronic wound, wound healing, synthetic, nanofiber, dressing, skin, wound material

## Abstract

Wound matrix materials are used to improve the regeneration of dermal and epidermal layers in both acute and chronic wounds. Contemporary wound matrices are primarily composed of biologic materials such as processed xenogeneic and allogeneic tissues. Unfortunately, existing biologic wound matrices possess multiple limitations including poor longevity, durability, strength, and enzymatic resistance required for persistent support for new tissue formation. A fully-synthetic, resorbable electrospun material (Restrata Wound Matrix, Acera, St.Louis, Missouri ) that exhibits structural similarities to the native extracellular matrix offers a new approach to the treatment of acute and chronic wounds. This novel matrix is the first product to combine the advantages of synthetic construction (e.g. resistance to enzymatic degradation, excellent biocompatibility, strength/durability and controlled degradation) with the positive attributes of biologic materials (e.g. biomimetic architecture similar to human extracellular matrix (ECM), fibrous architecture optimized to support cellular migration and proliferation, engineered porosity to encourage tissue ingrowth and vascularization). These features allow RWM to achieve rapid and complete healing of full-thickness wounds that, in preclinical studies, is comparable to Integra Bilayer Wound Matrix (Integra LifeSciences, Plainsboro, New Jersey), a gold standard biologic material with diverse clinical indications in the wound care. Together, this review suggests that the RWM offers a unique fully-synthetic alternative to existing biologic matrices that is effective, widely available, easy to store, simple to apply and low cost.

## Introduction and background

**Figure 1:**
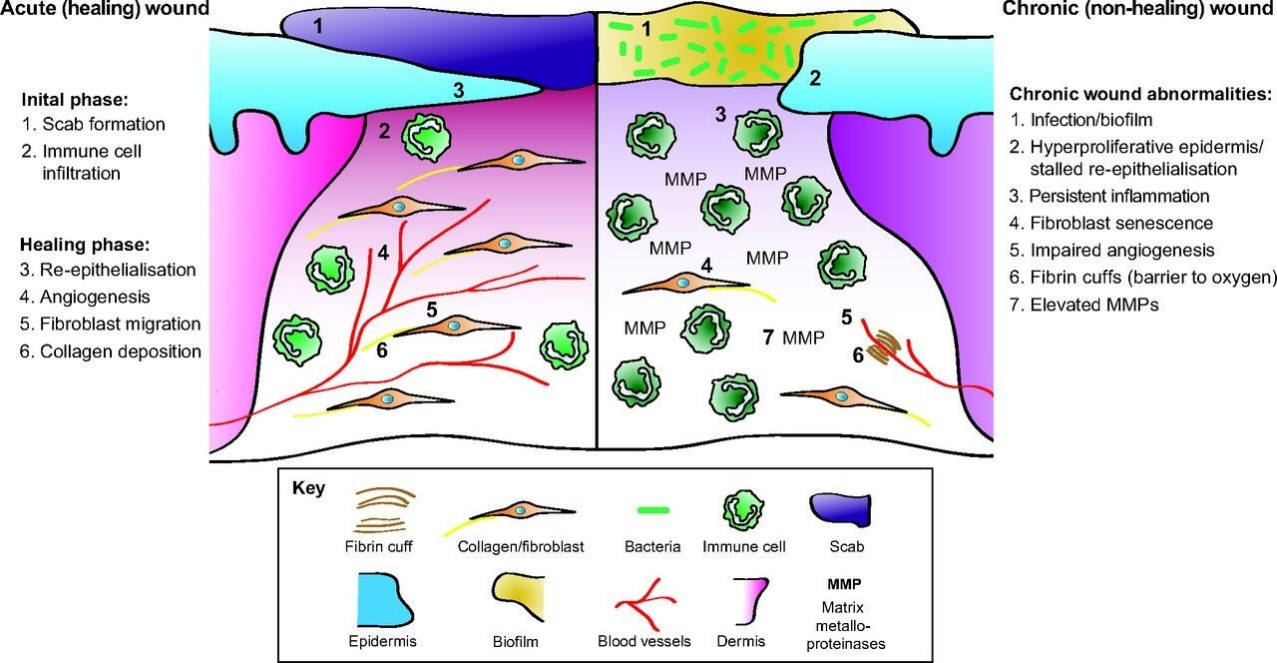
The diagram of processes governing normal (acute) wound healing and failed (chronic) wound healing. [5]

The wound healing is a complex process involving the participation of multiple cell types in the reconstruction of functional tissue. Normal wound healing proceeds in four stages: hemostasis, inflammation, proliferation, and remodeling [1-3]. Acute wounds are resolved by normal wound healing processes in a timeframe appropriate to the severity of the wound. In contrast, healing is delayed in chronic wounds due to the persistent trauma, infection, or ischemia as well as underlying conditions such as vascular disease or diabetes (Figure 1) [3]. Chronic wounds experience prolonged inflammation that is characterized by an abundance of enzymes in the wound that is unregulated due to depressed levels of their inhibitors [4]. Extracellular matrix (ECM) components, including newly synthesized collagen, are degraded by high levels of matrix metalloproteinases (MMPs), which thereby prevent repair of the wound tissue. Furthermore, increased levels of serine proteases can also degrade and thereby inactivate growth factors that would otherwise recruit the cell types necessary to progress wound healing beyond the inflammatory phase [2]. Chronic wounds stalled in an inflammatory state, thus cannot proceed to normal healing. This is a review article of the material technologies that currently exist to address chronic wounds, which also discuss what makes for an optimal wound matrix and subsequently introducing a novel synthetic nanofabricated scaffold, considering as a new technological space.

## Review

Common materials for healing chronic wounds

The engineered wound matrices have been developed to play an active role in healing chronic wounds [6-7]. Contemporary wound matrix materials are primarily composed of biologic materials and/or substrates. Many wound matrix products, such as Matriderm (MedSkin Solutions, Billerbeck, Germany) are composed of intact bovine collagen with or without another prevalent extracellular matrix (ECM) proteins. Collagen serves as a natural substrate for cellular attachment and thus supports tissue ingrowth, yet its degradation products can negatively influence cell migration and proliferation [8]. Processed collagen may also suffer from accelerated degradation due to the ease in which it can be broken down by matrix metalloproteinases (MMPs), as compared to native collagen [9]. Due to the foreign source of these biologic materials, collagen will always carry the risk of disease transmission, inflammatory response, and infection. For example, Integra Bilayer Wound Matrix (Integra LifeSciences, Plainsboro, New Jersey) is commonly utilized to treat partial and full thickness wounds, burn wounds, and chronic wounds [10-12]. Yet, Integra Bilayer Wound Matrix has demonstrated a 25% failure rate and requires the use of a skin graft to epithelialize some wounds [3,6]. Existing biologic wound matrices may also incorporate cellular components to enhance wound healing. Biologic matrices, such as Apligraf (Organogenesis, Canton, Massachusetts) is composed of bovine collagen matrix cultured with human fibroblasts meant to support the regeneration of the dermal and epidermal skin layers. In contrast, OrCel (Ortec International Inc, New York, United States of America) consists of a bi-layered material composed of cultured human fibroblasts in a cross-linked bovine collagen sponge layer and cultured keratinocytes in a collagen gel layer. This class of wound materials incorporating a cellular component often requires increasingly complex manufacturing techniques and has a limited shelf life.

Fully synthetic materials as a solution to common material limitations

Fully synthetic wound matrix materials can offer increased control of material and mechanical properties and avoid the risk of disease transmission, yet they commonly lack native tissue architecture and suitable biocompatibility profiles compared to biologic matrices. Synthetic materials include simple impervious dressings, such as Tegaderm (3M, Maplewood, Minnesota) and Opsite (Smith & Nephew, Andover, Massachusetts) which serve as a temporary physical barrier to protect the wound from trauma, infection, and dehydration. Alternatively, Bio-A Wound Matrix (Gore Medical, Flagstaff, Arizona) is a fibrous resorbable material composed of polyglycolic acid and trimethylene carbonate whose porous structure supports cellular infiltration, tissue ingrowth, and vascularization [13]. Bio-A has been shown to achieve greater tissue ingrowth, collagen deposition, and vascularization while inducing less inflammation than a biologic wound matrix and decreases the cost [13-14].

Composite biologic and synthetic materials have also been used to create wound matrices that simultaneously support tissue ingrowth and act as a temporary epidermis for mechanical protection and exudate control. Transcyte (Shire, Zug, Switzerland), for example, incorporates neonatal fibroblasts in a nylon mesh, where the mesh provides support for tissue ingrowth while the fibroblasts produce new ECM as well as regenerative factors such as cytokines [15]. Neonatal human fibroblasts have also been incorporated in fully resorbable synthetic matrices such as Dermagraft, composed of polyglactin mesh.

Optimal specifications for wound matrix materials

Existing wound matrix materials do not offer a solution capable of meeting all demands for clinical use. As such, there is a continued need for new wound matrix materials that can further improve outcomes for wound healing. An ideal wound matrix should avoid dehydration, prevent infection, and elicit minimal inflammation [16]. Additionally, an optimal wound matrix should be durable, such that it can provide long-lasting support of wound healing, by withstanding mechanical forces while maintaining flexibility. A wound matrix should temporarily reproduce the function of both dermis and epidermis during wound healing and encourage the complete restoration of both tissue layers to avoid wound contraction and scarring [6,17]. Furthermore, a wound matrix should be readily available for use, such that it can be applied immediately to wounds as needed. To be widely accessible, the wound matrix should also have a long shelf-life, simple storage requirements and low cost [6].

A novel synthetic nanofabricated scaffold: An introduction and review

Composition and Characteristics Overview

**Figure 2:**
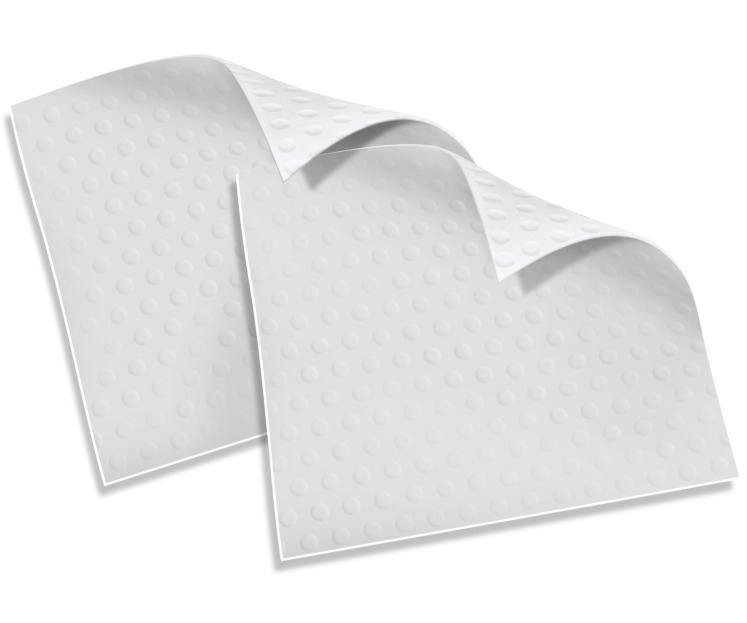
The Restrata Wound Matrix, a non-woven fully-resorbable material optimized for the wound care applications.

A novel synthetic nanofabricated scaffold (Restrata Wound Matrix (RWM), Acera Surgical, St. Louis, Missouri) has been developed which is a sterile, single-use device intended for use in the local management of wounds. The RWM is a soft, white, conformable, non-friable, absorbable matrix that acts as a protective covering for wound defects, providing a moist environment for the body’s natural healing process to occur (Figure 2). The RWM is made from synthetic biocompatible materials and was designed to include a fibrous structure with high porosity, similar to native ECM. The RWM is a porous matrix with a defined rate of resorption that provides a scaffold for cellular infiltration and vascularization before completely degrading via hydrolysis. The device permits the ingress of cells and soft tissue formation in the defect space/wound bed. The device does not contain any human or animal materials or tissues. The fibers are produced using a fully validated commercial scale electrospinning process. Thus, fiber elements whose scale and topography resemble native ECM are achieved with high reproducibility [16].

The RWM is composed of two synthetic polymers, polyglactin 910 poly (lactic-co-glycolic acid) (PLGA) (10:90) and polydioxanone, which are approved by the Food and Drug Administration (FDA) and currently utilized in biomedical applications such as resorbable sutures. These polymers are co-spun into non-woven sheets that are roughly 0.5 mm thick. This wound matrix is fully resorbable, as polyglactin 910 and polydioxanone are naturally degraded by hydrolysis.

Electrospinning creates a material that is soft and compliant, providing high conformability to wound surfaces, while being capable of withstanding clinician handling. Electrospun PLGA, for example, can obtain a tensile modulus of approximately 40-80 MPa, which mimics the tensile modulus of native skin [18]. The RWM also achieves adequate suture retention, such that it is suturable if necessary for securing the wound site. These mechanical properties also contribute to RWM’s durability, such that, this material can effectively protect the wound until appropriate healing has been achieved.

Restrata Wound Matrix Method of Action

The architecture, surface topography, and structural scale of wound matrices have a significant effect on the biological activity of regenerative cell populations [19]. The RWM possesses a fully-synthetic nanoscale architecture that enables unique material and biologic properties ideally suited to wound healing scenarios. These unique attributes of RWM are intended to support wound healing by encouraging tissue regeneration, neovascularization, and epithelialization.

Restrata Wound Matrix Exhibits Structural Attributes Similar to Native Extracellular Matrix Architecture

**Figure 3:**
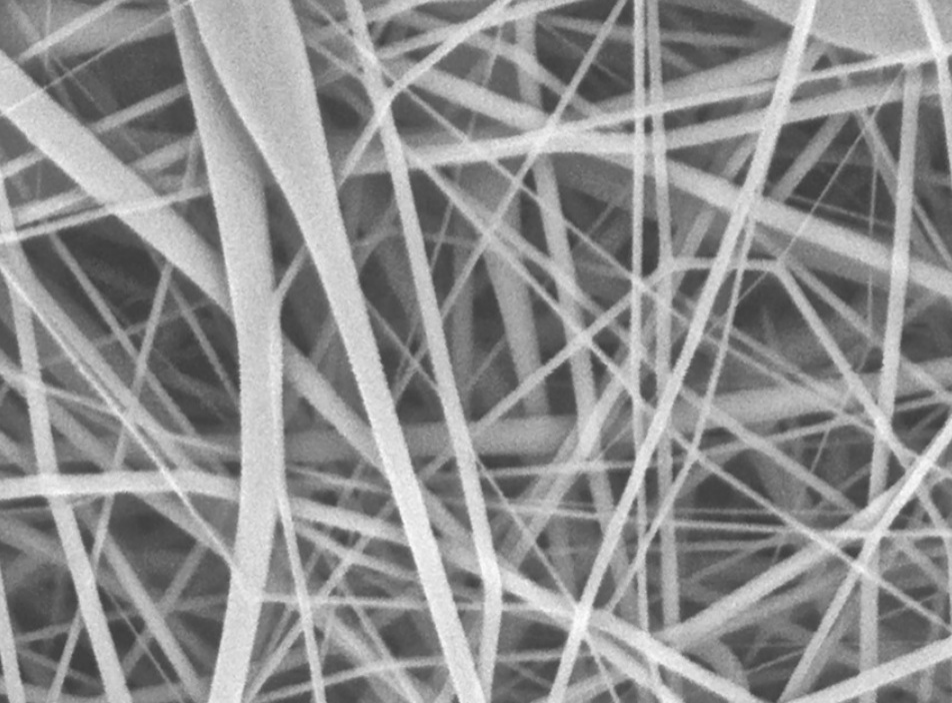
The Restrata Wound Matrix is a fully synthetic nanofabricated material consisting of a non-woven “fleece” composed of nanoscale polymer fibers.

The RWM is constructed of a blend of electrospun synthetic nanofibers that are similar to the structure of native ECM (Figure 3). In native skin, ECM is composed of non-woven collagen fibers approximately 50-500 nm in diameter [20]. In electrospun materials, the size of the polymer fibers can be tuned by parameters such as polymer concentration, polymer flow rate, and applied voltage [21]. Electrospinning, therefore, allows for the reproducible production of fibers that simulate the size and organization of natural ECM fibers [16]. The RWM is composed of non-woven synthetic fibers with a mean fiber diameter < 2000 nm and therefore mirrors the fiber size and organization of native ECM. Due to the unique biomimetic architecture, non-woven electrospun materials similar to RWM have been successfully used in the engineering of bone, skin, and blood vessels [21].

Electrospun Fibers Support Cellular Ingrowth, Differentiation and Retention

Electrospun fibers have been shown to support cellular ingrowth and migration critical to successful wound healing. Fibroblasts have been shown to proliferate and spread more rapidly and upregulate collagen expression on PLGA fibers 350-1100 nm in diameter, as compared to fibers of smaller or larger dimensions [18,22]. The RWM’s non-woven synthetic fibers range is from approximately 500 nm to 1500 nm and is therefore ideally positioned to facilitate cellular proliferation and activation.

Cell spreading and migration have also been influenced by the diameter of electrospun fibers. Synthetic fibers 350-1100 nm in diameter have been shown to affect the spreading, migration, and phenotype of adherent fibroblasts and mesenchymal stem cells (MSCs) [19,22]. The RWM’s non-woven synthetic fibers are ideally positioned in this range of fiber diameters in order to support cellular migration into the matrix. These findings further suggest that after progressive resorption of RWM’s nanoscale fibers in vivo, remaining microfibers (> 1000 nm) within the matrix, serve to retain infiltrating cell populations. Therefore, RWM’s unique blend of synthetic electrospun nano- and micro-fibers may support both rapid cellular ingrowth, migration, and activation, as well as continued cell retention and proliferation. Prior studies further suggest that electrospun fibers may have similar roles in encouraging the proliferation, migration, and differentiation of MSCs within the matrix [22].

The Porosity of Electrospun Matrix Supports Cellular Infiltration and Neovascularization

The RWM is comprised of an electrospun fiber matrix with engineered porosity suitable for both cellular ingrowth and neovascularization. Electrospun fibrous materials are capable of attaining an interconnected pore structure that is similar to natural tissue and permits cellular migration and tissue ingrowth as well as transport of oxygen, nutrients, and waste [21]. The high surface area afforded by the nanoscale fibers provides a substantial substrate for cell attachment, oxygen permeation, and the management of exudate [17-18]. The pore size and degree of porosity are also critical in facilitating cell migration and vascularization [19,22]. Highly porous matrices (60-90% porous) are advantageous for cellular infiltration and tissue ingrowth, while pore sizes of 90-130 μm are sufficient to permit fibroblast migration and proliferation [23-24]. Furthermore, pore sizes of 5-500 µm diameter have been shown to facilitate the successful invasion of new vasculature into the construct’s interior, the absence of which contributes to cell death and tissue necrosis [22]. The porosity of RWM’s non-woven synthetic fiber matrix is ideally positioned within this range in order to support cellular ingrowth and neovascularization. Furthermore, gradual resorption of RWM’s nanoscale fibers following in vivo application results in a progressive increase in matrix porosity. A continuous increase in matrix porosity supports increasing tissue formation and vascularization within the electrospun matrix and a controlled transition of mechanical loading from the synthetic wound matrix to the newly formed tissue.

Fully-Synthetic Construction Offers Excellent Biocompatibility

The synthetic composition of RWM offers a suitable scaffold for cellular ingrowth and tissue formation without the need or use of biologic xenogeneic or allogeneic materials or substrates. The RWM, therefore, offers a wound matrix material devoid of any risk of allergic response to animal products, zoonotic disease transmission, or ethical/religious concerns. The RWM’s use of well-characterized medical grade synthetic polymers that exhibit excellent biocompatibility can also significantly reduce the inflammatory response at the site of application. Compared to the inevitable host response elicited by biological materials from foreign human (allograft) or animal (xenograft) sources, electrospun wound matrix materials may offer reduced levels of inflammation within the wound bed both at acute and sub-acute timepoints. The RWM is also fully resorbable, similar to leading biologic xenograft and allograft products. The resorbable nature of RWM ensures that no permanent implant or material will remain at the site of application and that no chronic inflammatory reaction against the material will exist to limit wound healing or resolution.

Fully-Synthetic Construction Offers Resistance to Enzymatic Degradation 

Resorption of RWM’s electrospun polymer matrix occurs in controlled stages that are governed by progressive hydrolysis rather than rapid enzymatic proteolysis. The RWM is composed of multiple electrospun polyglactin 910 and polydioxanone fiber populations. The RWM’s synthetic electrospun fiber matrix is therefore resistant to key enzymes released by inflammatory cells (e.g. MMPs) within the wound bed. The RWM thereby resists rapid enzymatic degradation at the wound site, avoiding premature degradation by proteases that directly contribute to and are overexpressed in chronic non-healing wounds. Wound matrices such as RWM that can resist protease degradation can provide a more stable and a more persistent scaffold capable of supporting wound healing over a longer time course [25].

Increased Persistence in Wound Bed May Lead to Fewer Re-Applications

The RWM has been engineered to resorb at a rate that matches the time course of tissue ingrowth. Due to its polymeric composition, RWM gradually resorbs via hydrolysis within the wound bed over the course of roughly 30 days. This prolonged presence within the wound site is anticipated to provide continued support of cellular infiltration, vascularization, and re-epithelialization, which occurs over a course of three weeks in normal wound healing [26]. The time-scale over which RWM is present within the wound bed is significantly longer than existing biologic scaffolds currently in the clinical use. The longevity of RWM within the wound site may enable a longer period of wound healing and cellular ingrowth, and possibly eliminate the need for continued reapplication over the course of treatment. Thus, the maintenance of the wound site may only require changing outer wound dressings, rather than the wound matrix, and reduce the number of painful procedures experienced by the patient. Furthermore, complete resorption of RWM precludes any persistent chronic foreign body response.

Degradation Products Elicit Antimicrobial Effect

While RWM does not contain any active agents or drugs, the RWM is well suited for use in colonized wounds. Previous studies have demonstrated that the monomers and degradation products of RWM have antimicrobial effects [22]. For example, lactic acid/polylactic acid have been shown to have an antimicrobial effect across multiple bacterial strains [27-28]. The initial pore size of RWM’s non-woven electrospun scaffold falls within the range proven to prevent bacterial penetration (1–10 μm) [17]. This suggests that bacteria will have a more difficult time penetrating the matrix and contaminating the wound. The nanofiber matrix of RWM also permits infiltration of inflammatory cells necessary to remove any bacteria/pathogens present within the electrospun matrix. The RWM’s fully-resorbable construction also suggests that the entire matrix can be cleared without the risk of biofilm formation typical of permanent implants. Fully-synthetic RWM is also resistant to enzymatic degradation and is not affected by bacteria in the wound bed, unlike collagen-based biologic materials.

Intended Use of Restrata Wound Matrix

The RWM is intended for use in the management of wounds, including: partial and full thickness wounds, pressure sores/ulcers, venous ulcers, diabetic ulcers, chronic vascular ulcers, tunneled/undermined wounds, surgical wounds (e.g. donor sites/grafts, post-laser surgery, post-Mohs surgery, podiatric wounds, wound dehiscence), trauma wounds (e.g. abrasions, lacerations, partial thickness burns, skin tears), and draining wounds. The RWM is not indicated for use in third-degree burns and should not be used in the patients with known sensitivity to resorbable suture materials.

The RWM is supplied in the form of terminally sterile, in a single use double peel package in a variety of sizes. The contents of the package are guaranteed sterile and non-pyrogenic unless the package has been opened or damaged. The RWM is available in sizes ranging from 2.5cm x 2.5cm up to 12.5cm x 17.5cm.

Preclinical Demonstration of Restrata Wound Matrix in a Porcine Wound Healing Model

**Figure 4:**
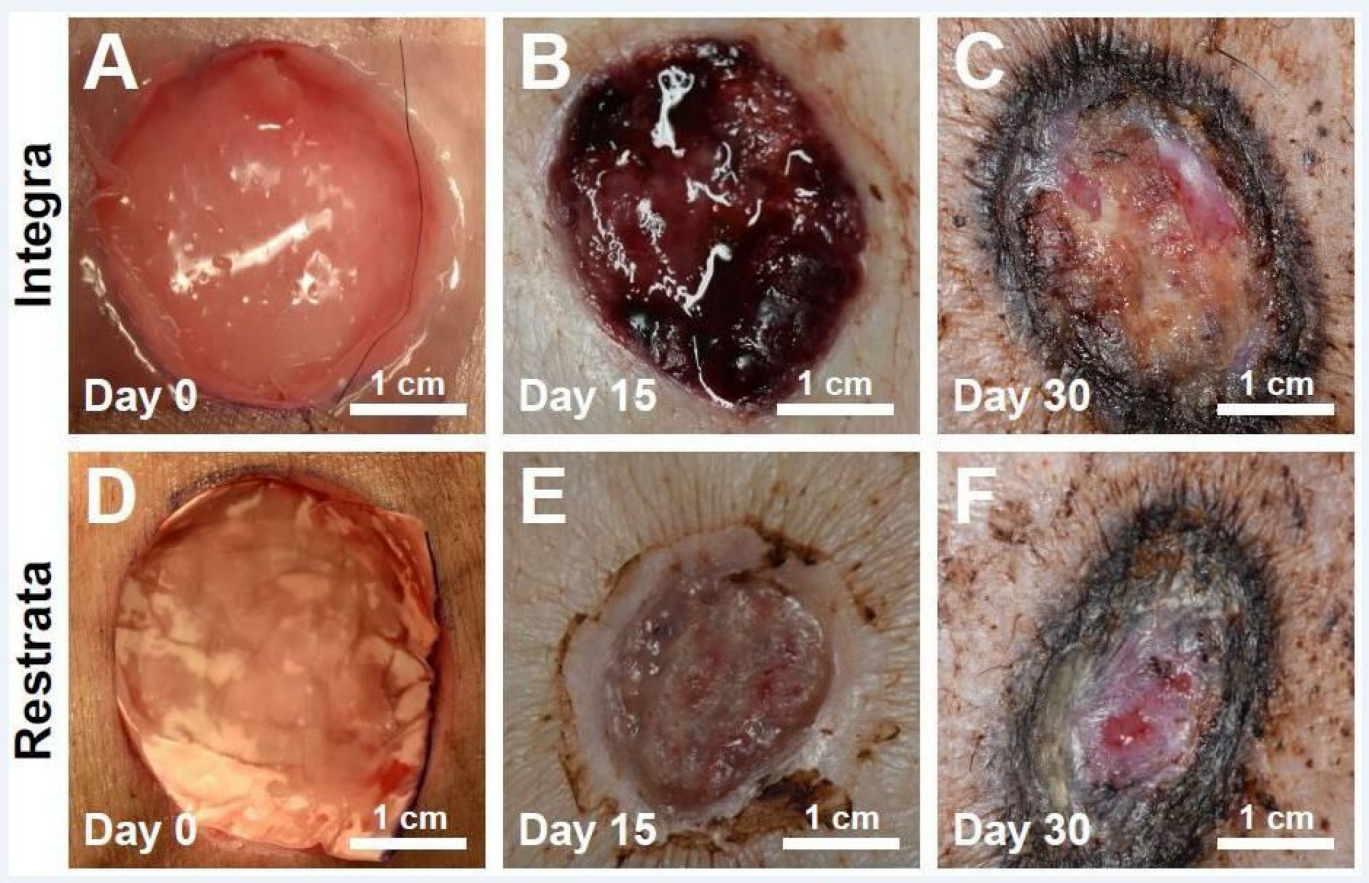
Full-thickness cutaneous wounds immediately after the application at day 15 and day 30 for Integra® Bilayer Wound Matrix (control-A, B, C) or Restrata Wound Matrix (treatment-D, E, F).

The RWM was compared to the Integra Bilayer Wound Matrix in the treatment of full-thickness cutaneous wounds in Yucatan miniature swine. Full thickness wounds (3 cm in diameter) were created along the dorsum of the swine and the Integra Bilayer Wound Matrix or the RWM was applied directly to the wounds (Figure 4).

**Figure 5:**
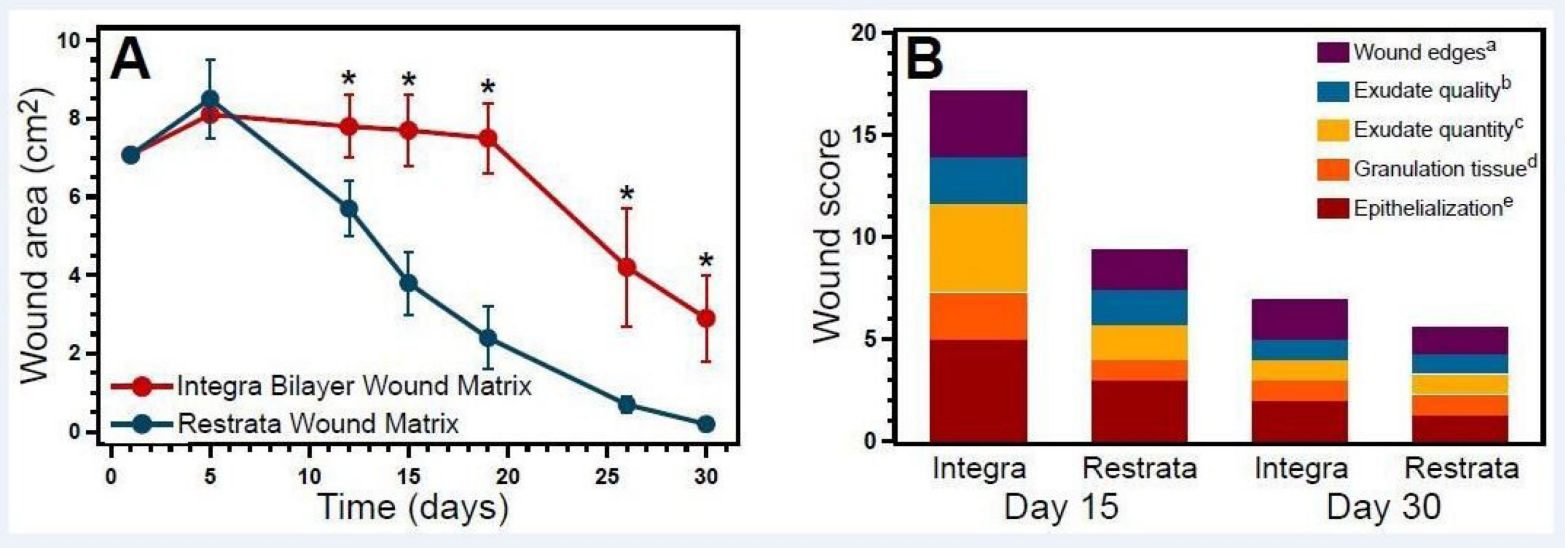
Gross evaluation of the inflammation and wound healing. A) Wound area (average ± standard deviation (SD) as determined by planimetric analysis of the wound photographs, where *p

Both RWM and the Integra Bilayer Wound Matrix supported wound healing, however, wound area decreased more rapidly with RWM (Figure 5A). Compared to measurements made on day five, wounds treated with RWM decreased wound area by 98% (on an average) by day 30, while wounds treated with Integra Bilayer Wound matrix decreased only by 64%. Furthermore, wounds treated with RWM were smaller than wounds treated with Integra Bilayer Wound Matrix at each time following day five. Two of the three wounds treated with RWM were fully healed by Day 30 (wound area = 0 cm^2^). Under the conditions of this study, RWM sped wound closure compared to Integra Bilayer Wound Matrix.

The wounds treated with RWM also had an average Bates-Jensen wound score that was 46% and 19% lower than for wounds treated with the Integra Bilayer Wound Matrix by day 15 and day 30, respectively (Figure 5B). The wounds treated with RWM had more granulation tissue by day 15 and greater epithelialization at both the days 15 and 30 than the wounds treated with the Integra Bilayer Wound Matrix.

**Figure 6:**
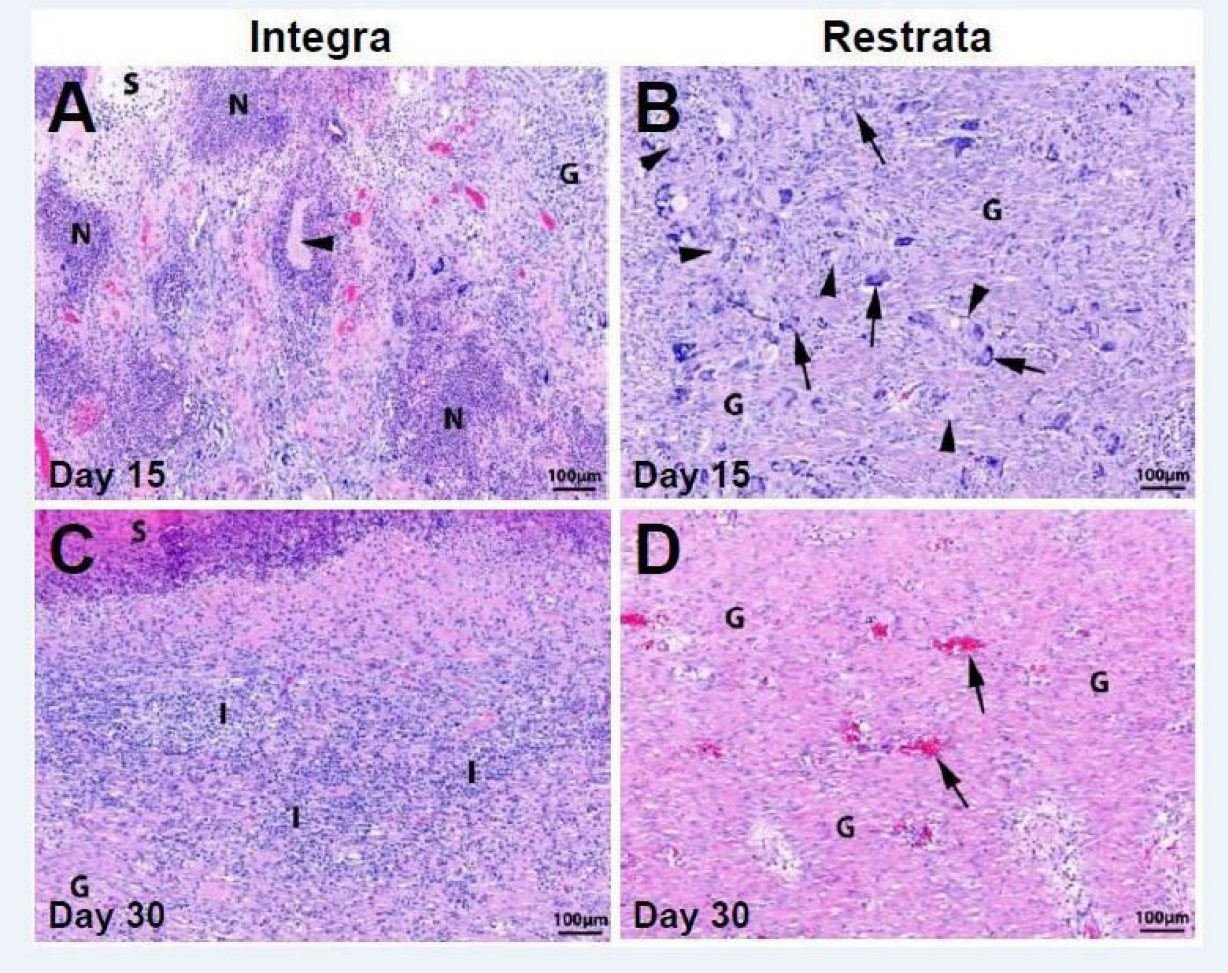
Hematoxylin and eosin stained sections from wounds treated with (A) Integra® Bilayer Wound Matrix or (B) Restrata Wound Matrix at Day 15. The G represents the granulation tissue, N: neutrophils, S: seroma, arrowheads showing the wound matrix material, arrows showing the multinucleated giant cells surrounding wound matrix material. Hematoxylin and eosin stained sections from wounds treated with (C) Integra® Bilayer Wound Matrix or (D) Restrata Wound Matrix at Day 30. The G represents the granulation tissue, I: inflammation (infiltrating neutrophils and macrophages), S: serocellular debris. Arrows showing the blood vessels.

Histological analysis of wound tissue sections revealed significant differences in inflammation, granulation tissue, collagen maturation, vascularization, and epithelialization of wounds treated with either material (Figure 6). The RWM encouraged more rapid ingrowth of granulation tissue, as compared to the Integra Bilayer Wound Matrix. The granulation tissue completely covered the wounds treated with RWM at day 15, while only 20-50% of the wound area treated with Integra Bilayer Wound Matrix was covered with granulation tissue at this time point. The RWM also encouraged more rapid deposition and maturation of collagen. Vascularization was also accelerated by RWM, as compared to the Integra Bilayer Wound Matrix, with the greater presence of neovasculature noted at day 15. Finally, epithelialization was more rapid and complete in wounds treated with RWM. Under the conditions of this study, RWM exhibited improved biocompatibility and wound healing compared to the Integra Bilayer Wound Matrix.

## Conclusions

No existing clinical materials meet all the expectations of an ideal regenerative wound matrix. Current options for wound care range from graft materials, including autografts, allografts, and xenografts, to engineered materials, are derived from biologic or synthetic components. Traditionally, synthetic wound matrix materials have offered excellent control over material and mechanical properties, but have lacked the architecture and regenerative properties of biologic matrices.

Restrata wound matrix (RWM), a fully-synthetic, resorbable electrospun material that possesses structural elements similar to natural ECM, offers a new approach to the treatment of acute and chronic wounds by combining the advantages of synthetic construction with the positive attributes of biologic materials. Further characterization of the novel RWM material in the pre-clinical and clinical settings will be showing the breadth of application.
